# The role of Collagen Tissue Scaffolds in 3D Endometrial-like Culture Systems: Important Contributions to Cell Invasion and Cell Topography

**DOI:** 10.1007/s43032-025-01800-2

**Published:** 2025-02-05

**Authors:** Remziye Kendirci-Katirci, Aylin Sendemir, Elif Esin Hameş, H. Seda Vatansever

**Affiliations:** 1 Department of Histology and Embryology, School of Medicine, Akdeniz University, Antalya, Türkiye; 2 Department of Bioengineering, Faculty of Engineering, Ege University, Izmir, Türkiye; 3 Department of Histology and Embryology, School of Medicine, Manisa Celal Bayar University, Manisa, Türkiye; 4 DESAM Research Institute, Near East University, Mersin 10, Türkiye

**Keywords:** 3D cell culture techniques, Bacterial cellulose, Collagen, Epithelial-mesenchymal transition, Embryo implantation

## Abstract

Considering the similarity between the invasion processes of cancer cells and embryo implantation, three-dimensional culture models used to study cancer cell invasion can also be applied to embryo implantation studies. In our study, endometrial epithelial cell line (RL95-2) and spheroid-forming trophoblast-like choriocarcinoma cell line (JAR) were cultured on three different biocompatible tissue scaffolds: bacterial cellulose, collagen foam and collagen fibre. These scaffolds are frequently used in cancer cell metastasis and invasion studies, A three-dimensional endometrium-like culture system was established to quantitatively investigate the role of E-cadherin, N-cadherin, Vimentin, α-smooth muscle actin and Syndecan-1 proteins in the type 1 epithelial mesenchymal transition mechanism observed during the invasion step of the implantation process. Based on the findings from the three-dimensional cell culture, the bacterial cellulose scaffold promoted the proliferation of RL95-2 cells and delayed JAR spheroid formation. The collagen foam scaffold favored the proliferation of RL95-2 cells and accelerated JAR spheroid formation. The collagen fibre scaffold is important for supporting cell topography and, when combined with collagen foam, may offer a potential solution for investigating 3D endometrium-like culture systems. Immunocytochemical and immunofluorescence analyses showed that scaffolds modulate the invasion process by affecting the expression of epithelial mesenchymal transition proteins in cells. The findings suggest that different tissue scaffolds can produce varying effects in endometrium-like culture systems, and combinations of these materials may yield more effective results in future studies. This research represents a critical step in studying cell behavior in 3D culture systems and elucidates the mechanism of endometrial invasion.

## Introduction

Implantation is a physiological event in which the embryo and endometrium interact. During implantation, the process of invasion from epithelium to mesenchymal tissue begins, which is defined as type 1 epithelial mesenchymal transition and subsequently supports a healthy pregnancy [1, 2]. Since many placental and pregnancy complications are related to implantation, it is very important to elucidate the interactions and invasion mechanisms of the endometrium and trophoblast, which are necessary for the establishment of a healthy pregnancy [3].

Embryo implantation research is of great importance in three-dimensional culture systems that provide mechanical support for cell attachment and help maintain tissue integrity [4]. Bacterial cellulose (BC) is biocompatible and promotes cell proliferation. Biocompatible collagen foam (COL/FO) and collagen fibre (COL/FI) tissue scaffolds that support tissue regeneration and promote cell invasion through their cross-links are also widely used three-dimensional (3D) culture models. These models, especially in cancer cell invasion and metastasis research, allow cells to invade with the cross-links they contain [5, 6]. Considering that the invasion steps of cancer cells and embryo implantation process are similar, 3D culture models used to investigate the invasion steps of cancer cells are also an opportunity for embryo implantation research [7]. Especially, considering the difficulty of working with primary human cells and ethical problems, it is of great importance to investigate the human implantation process in 3D endometrium-like culture systems to be established [8].

In this study, it was aimed to develop a 3D endometrium-like culture system that can be used in embryo implantation model and to investigate the mechanism of type 1 epithelial mesenchymal transition in this process. For this purpose, it was aimed to establish a 3D endometrium-like culture system with endometrial epithelial cell line (RL95-2) and spheroid-forming trophoblast-like choriocarcinoma cell line (JAR) on BC, COL/FO and COL/FI tissue scaffolds and then to investigate the type 1 epithelial mesenchymal transition mechanism seen in the invasion step of the implantation process.

## Materials and Methods

### Cell Culture

The endometrial epithelial cell line RL95-2 (CRL-1671) (ATCC-58027481) was purchased from ATCC and cultured in Dulbecco’s Modified Eagle Medium (DMEM): F12 (Biosera, LM_D12221500) culture medium supplemented with 10% FBS (Capricorn Scientific, FB_100H/100), 1% penicillin-streptomycin (Capricorn Scientific, PS-B) and 1% L-glutamine (Capricorn Scientific, GLNB).

The spheroid-forming trophoblast-like choriocarcinoma cell line JAR (HTB-144) (ATCC-59061047) was purchased from ATCC. The culture medium including 10% fetal bovine serum (FBS), 1% penicillin-streptomycin, 1% L-glutamine and RPMI-1640 (Biosera, LM_R16391500) was used for culture of JAR cells. All culture conditions were standardized at 37Cº with 5% CO_2_ in incubator (ESCO, CCL-170B-8). The medium was changed every two days and the cells were observed under an inverted microscope (Olympus, IX71).

### Production of Tissue Scaffolds

Three different types of biocompatible tissue scaffolds were produced in the Animal Cell Culture and Tissue Engineering Laboratory and Medical Biotechnology Laboratory of Ege University, Department of Bioengineering.

*Gluconacetobacter xylinus* ATCC 700,178 was used for bacterial cellulose (BC) production. *G. xylinus* was activated in sterile Hestrin&Schramm (HS) liquid medium at 30 °C and 150 rpm for 1–2 days prior to production. The HS medium consists of distilled water, 2% glucose (MERCK 108347), 0.5% yeast extract (MERCK 103753), 0.5% bacteriological peptone (MERCK 107214), 0.115% citric acid (MERCK 100244) and 0.27% Na_2_HPO_4_ (MERCK 106580). The pellets formed after activation were inoculated into HS liquid medium containing shredded agar particles and incubated for 5 days in static culture conditions. Agar particles used to increase BC porosity were prepared with 5% (w/v) agar agar (Oxoid LP0011) and distilled water, sterilized and shredded in HS medium (1:1) using a hand blender under aseptic conditions after solidification [9]. The produced BC membranes were purified to remove nutrients and cell debris. Specifically harvested BC membranes were rinsed with distilled water five times and hold in 0.1 M NaOH (MERCK 106462) for overnight subsequently were boiled in fresh NaOH for 20 min. Then repeated washing with pure water until the pH was neutral and sterilised by autoclaving at 121 °C for 15 min.

Collagen foam (COL/FO) tissue scaffold production was performed by lyophilisation method. Collagen type 1 (Santa Cruz Biotechnology, sc136157) solution prepared at 5% in acetic acid was added to cell culture dishes (6–12 or 24 wells). The solution was kept at −20 °C for the production of large pore scaffolds and at −80 °C overnight for the production of small pore scaffolds. The frozen solution was lyophilised for 8 h to prepare COL/FO tissue scaffolds. The scaffolds were kept in a desiccator until used. Sterilisation with 70% ethanol solution (Sigma, 32221) was performed before use.

Collagen fibre (COL/FI) tissue scaffold production was performed by electrospinning method. 55 mg collagen (Santa Cruz Biotechnology, sc136157) was dissolved in 1 mL HFP (1,1,1,1,3,3,3,3- hexafluoro-2-propanol) (Sigma C9791) with a magnetic stirrer for 1 day. The prepared homogenous collagen solution was drawn into a 5 mL syringe and placed in the electrospinning device. The collector plate was covered with aluminium foil and the distance to the syringe tip was set as 15 cm. Lamellae with a diameter of 10 mm were glued on the aluminium foil and the pump was operated at 1 mL/sec and the electric current was 18 kV. Collagen fibre tissue scaffolds were spun on the coverslips. The scaffolds were UV sterilised for 120 min and prepared for use.

### Scanning Electron Microscopy (SEM)

The surface structures and pore diameters of the scaffolds were visualised by scanning electron microscopy (SEM). All scaffolds were lyophilised and dried after fabrication. Their surfaces were made conductive by coating with gold spraying before SEM (Carl Zeiss 300 VP) imaging.

### Establishment of a 3D Culture Model

BC, COL/FO and COL/FI tissue scaffolds were taken into 24-well culture dishes 1 day before seeding of cells and incubated with 500 µl culture medium without cells for 1 night. At the end of the period, RL95-2 and spheroidal JAR cells were seeded onto the tissue scaffolds and 3D culture models were created. For this purpose, 1 × 10^6^ RL95-2 cells/ml were seeded onto the tissue scaffolds. On these cells, 1.8 × 10^6^ JAR cells/ml were seeded. After cell seeding, the medium was changed every two days. The 3D culture models were fixed with 4% paraformaldehyde (Merck, TP704404) on the 3rd, 5th, 7th, 10th and 14th days of culture time and stored at + 4 Cº. The experiments were independently repeated three times (3 technical replicates) in three independent biological samples (*n* = 3).

### Frozen Section

BC and COL/FO scaffolds were embedded with OCT embedding medium (Jung, 020108926). at −20 Cº for 5–10 min, after then 7 μm sections were taken from each sample using Cryostat (Leica, CM1100). Sections were stored at −20 Cº until further investigation. COL/FI scaffolds could not be sectioned because they were on glass.

### Immunocytochemistry

Frozen sections were kept in room temperature for 24 h before immunocytochemistry staining protocol. They were washed with phosphate buffer solution (PBS) for 2 times for 30 min. For permeabilization, 0.1% Triton X-100 (Applichem, 4L003808) was added on slides onto ice for 15 min. Then the slides were washed with PBS for 3 times for 5 min and 3% H_2_O_2_ (Merck, K31355100303) added on them for 10 min at room temperature. The slides were washed again with PBS for 3 times for 5 min and incubated with blocking solution (Invitrogen, 859043) at room temperature for 1 h. At the end of the time, remove the blocking solution without washing step, primary antibodies anti-E-cadherin (Proteintech, 20874-1-AP), anti-N-cadherin (Proteintech, 13769-1-AP), anti-Vimentin (Vector, EB11207), anti-α-SMA (Novus, NB300-978), and anti-Syndecan-1 (Proteintech, 10593-1-AP) were applied in 1:100 dilution for each antibodies and incubated overnight at + 4 Cº. The slides were then washed with PBS for 3 times for 5 min and incubated at room temperature for 30 min with the addition of biotinylated secondary antibody. At the end of the time, the samples were washed with PBS again, and incubated streptavidin HRP antibody for 30 min at room temperetaure. They were washed with PBS and added daiminobenzidine (DAB) (Scytek, AEM080) for 5 min. The slides were then washed with distilled water for 3 times for 5 min and stained with Mayer’s Haematoxylin (72804E, Microm, Walldorf, Germany) for 2–3 min at room temperature. After washing in distilled water for 10 min, the slides were mounted with mounting medium (Spring bioscience-DMM125). The experiments were independently repeated three times (3 technical replicates) in three independent biological samples (*n* = 3). The intensity and distribution of E-cadherin, N-cadherin, Vimentin, α-SMA, and Syndecan-1 was measured using ImageJ software (NIH, Bethesda, MD, USA), and statistical analysis was performed using GraphPad Prism software 9.

### Double Immunofluorescence

Collagen fiber tissue scaffolds could not be sectioned because they were on glass and double immunofluorescence staining was performed directly. Samples were fixed with 4% paraformaldehyde for 30 min and washed with PBS for 30 min. Permeabilisation was performed with Triton X-100 solution containing 1% Bovine Serum Albumin (BSA, Sigma, A7906) on ice for 15 min. At the end of the time, the samples were washed 2 times for 2 min with PBS containing 1% BSA. At the blocking stage, the samples were incubated with 10% goat serum (Sigma, S26-M) prepared in PBS containing 1% BSA for 1 h at room temperature. Then anti-E-cadherin and anti α-SMA, anti-Vimentin and anti-N-cadherin and anti-Syndecan-1 antibodies were prepared in 1/100 dilutions and kept at room temperature for 1 h. At the end of the time, the samples were washed 2 times for 2 min with PBS containing 1% BSA. Then, 1/500 dilution of rabbit anti-goat FITC (Millipore, AP308F) for anti α-SMA, and anti-Vimentin antibodies or goat anti-rabbit Rodamine (Santa Cruz Biotechnology, sc-2091) for anti-E-cadherin, anti-N-cadherin and anti-Syndecan-1 antibodies was added to the samples and incubated at room temperature for 30 min. After washing with PBS for 3 times for 5 min, DAPI solution (Cell Signaling Technology 40835) was added for 10 min at room temperature for staining of nuclei. They were then mounted with mounting medium (Calbiochem, JA1750) which was suitable for fluorescent staining. The staining was examined under an immunofluorescence attachment microscope (Olympus IX71). The experiments were independently repeated three times (3 technical replicates) in three independent biological samples (*n* = 3). The mean fluorescence intensity of E-cadherin, N-cadherin, Vimentin, α-SMA, and Syndecan-1 was measured using Image J software (NIH, Bethesda, MD, USA), and statistical analysis was performed using GraphPad Prism software 9.

### Statistical Analysis

The experimental data were analyzed using descriptive statistics, including the calculation of means and the standard error of the mean. Normality tests were conducted using the Shapiro-Wilk test. Parametric data were analyzed using one-way ANOVA test and then compared between selected groups. The statistical comparisons were analyzed using the Sidak post hoc test. Non-parametric data were analyzed using Kruskal-Wallis test followed by post hoc Dunn’s test. Data are presented as mean ± SEM. The specific statistical tests used for each dataset are specified in the figure legends. GraphPad Prism software version 9 was employed for all statistical analyses, and statistical significance was set at a p-value < 0.05.

## Results

### 2D Cell Culture Result

**Fig. 1:**
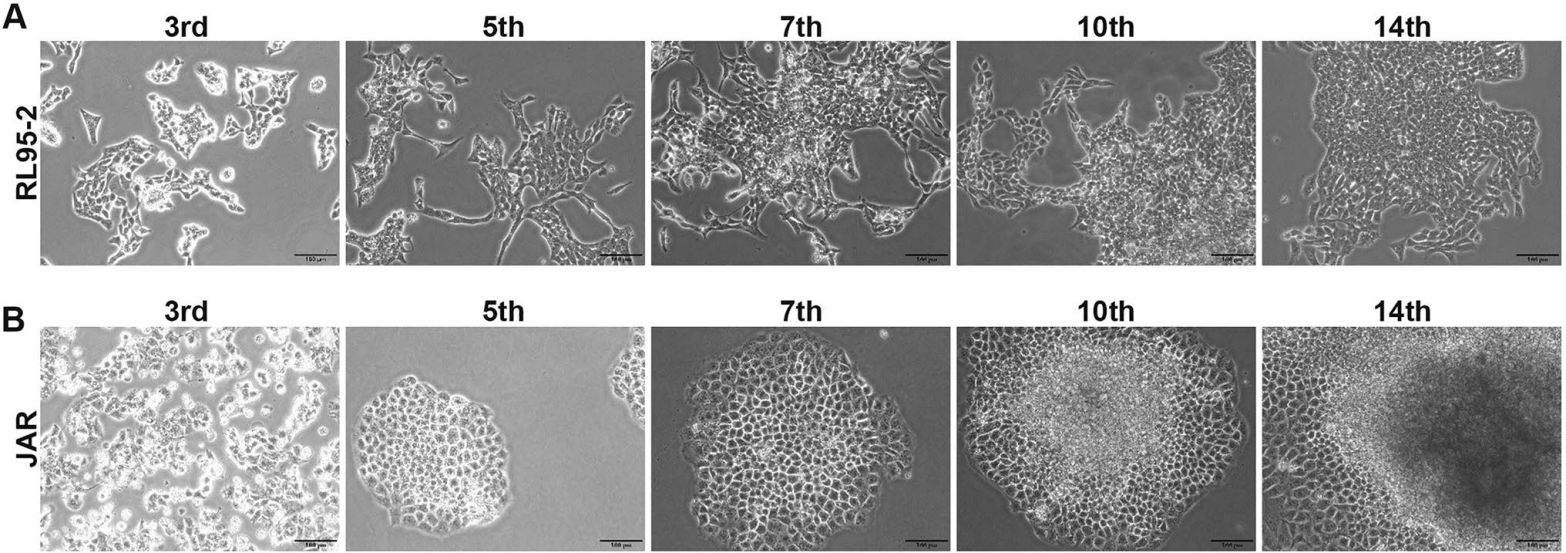
2D cell culture micrograph. **A** Micrograph of RL95-2 cells from different days of cell culture. **B** Micrograph of JAR cells from different days of cell culture. The scale bars:100 μm

The endometrial epithelial cell line (RL95-2), which tends to proliferate in a fusiform structure and monolayer, maintained its morphological features throughout the culture period (Fig. 1A). It has been observed that the trophoblast-like choriocarcinoma cell line (JAR) tends to cluster and form a spheroid structure starting from the 5th day of culture. It was observed that the cells continued to proliferate from the upper part of the spheroid structure from the 10th day of culture (Fig. 1B).

### Characterization Findings of Tissue Scaffolds by SEM

**Fig. 2:**
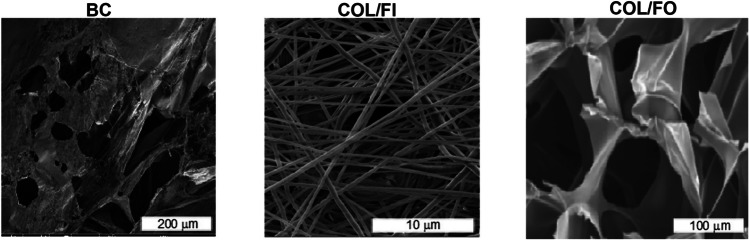
Characterization of tissue scaffolds with SEM. SEM images of bacterial cellulose (BC), collagen foam (COL/FO) and collagen fibre (COL/FI) scaffolds. Scale bars: 200 μm, 10 μm, 100 μm respectively

3D endometrium-like culture systems created with BC tissue scaffold tissue scaffolds were visualized with SEM and analyzed with the Image J program. The pore diameters of BC, COL/FI and COL/FO scaffolds are 97 ± 28 μm, 0.67 ± 0.14 μm, and 109 ± 21 μm, respectively (Fig. 2).

### 3D Cell Culture Result

#### 3D Endometrium-like Culture System Created With COL/FI Tissue Scaffold Strongly Supports Spheroid Formation in JAR Cells

**Fig. 3:**
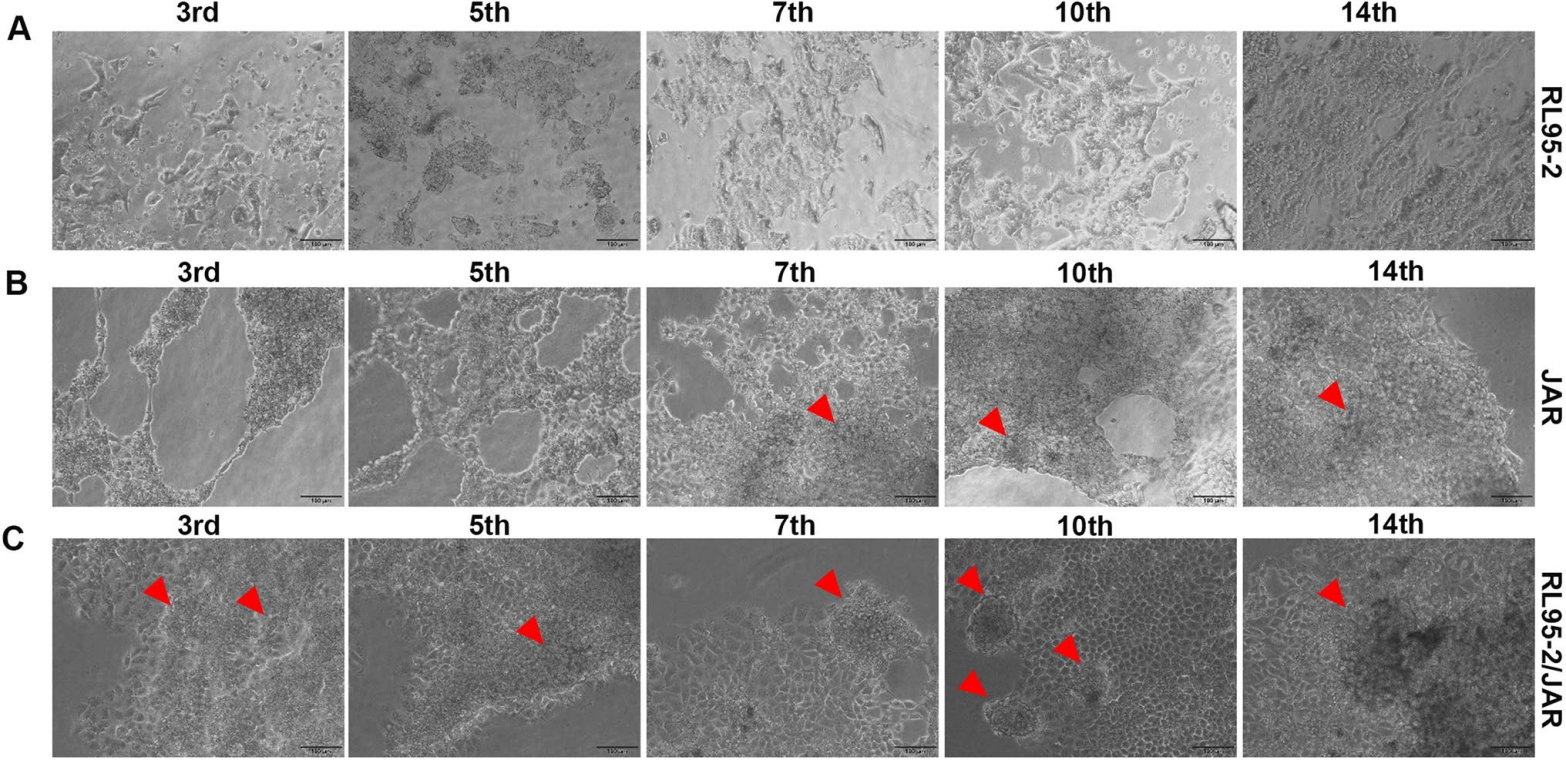
3D endometrium-like culture system created with BC scaffold. **A** Phase-contrast image of RL95-2 cells in BC scaffold from different days of culture. **B** Phase-contrast image of JAR cells in BC scaffold from different days of culture. **C** Phase-contrast image of RL95-2/JAR cells in BC scaffold from different days of culture. The red arrowhead shows spheroids formed by JAR cells. The scale bars:100 μm

In 3D endometrium-like culture systems created with BC tissue scaffold, differences were observed in the cells observed under an inverted microscope on the 3rd, 5th, 7th, 10th, and 14th days of cell culture time. Accordingly, in RL95-2 cells planted alone on the BC scaffold, it was observed that the cells continued to proliferate throughout all days of culture time (Fig. 3A). A spheroid structure was observed in JAR cells on the 7th day of culture firstly and an increase in the surface of the spheroids was detected when the culture continued (Fig. 3B). In the 3D endometrium-like culture system that was created by seeding RL95-2 and JAR cells together on the BC tissue scaffold, JAR cells formed their first spheroid structures on the 3rd day of culture, and when the culture continue it was increased (Fig. 3C).

**Fig. 4:**
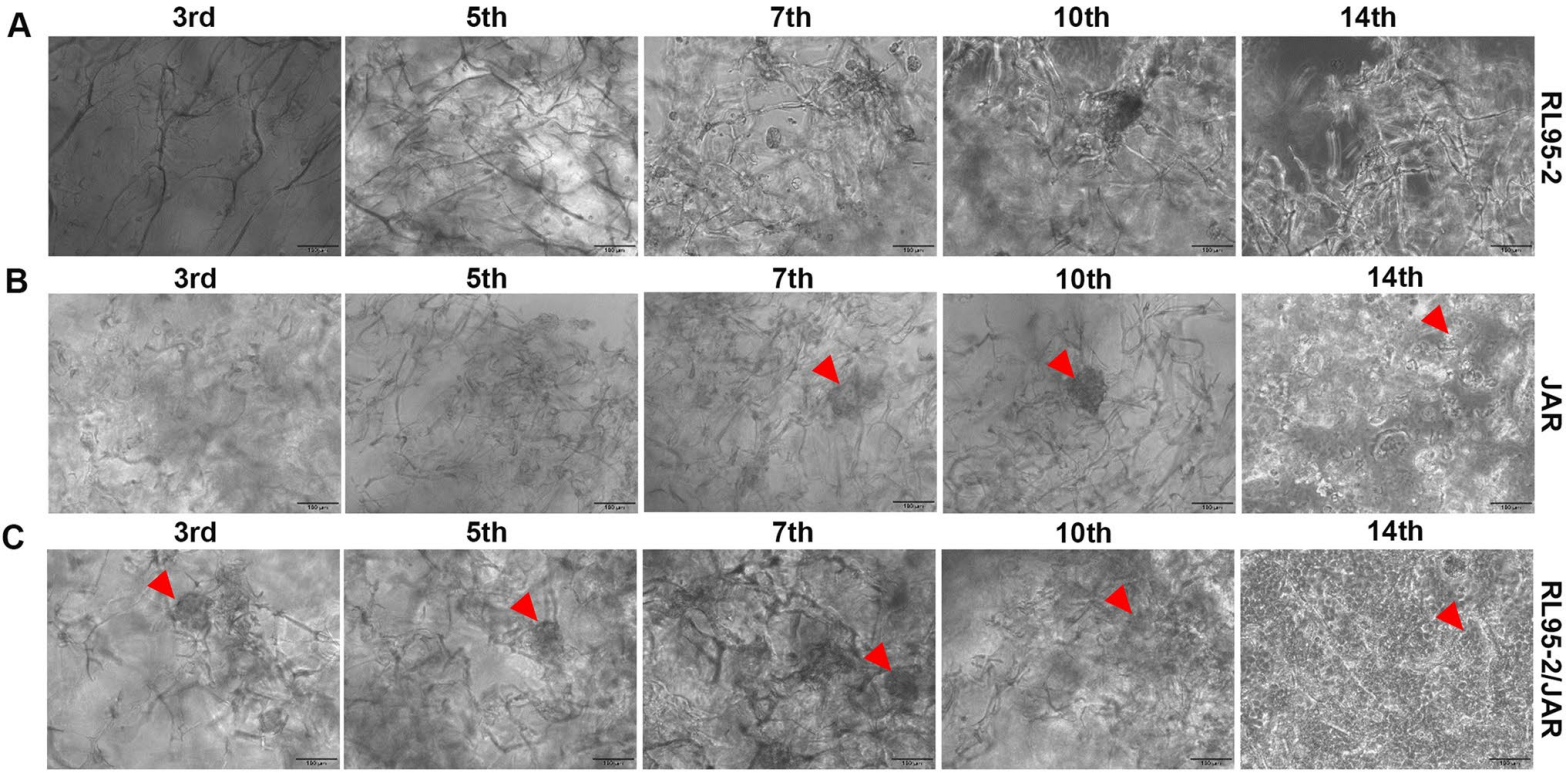
3D endometrium-like culture system created with COL/FO scaffold. **A** Phase-contrast image of RL95-2 cells in COL/FO scaffold from different days of culture. **B** Phase-contrast image of JAR cells in COL/FO scaffold from different days of culture. **C** Phase-contrast image of RL95-2/JAR cells in COL/FO scaffold from different days of culture. The red arrowhead shows spheroids formed by JAR cells. The scale bars:100 μm

In 3D endometrium-like culture systems created with COL/FO tissue scaffold, differences were observed in the cells examined under an inverted microscope on days 3rd, 5th, 7th, 10th, and 14th of culture time. Accordingly, in RL95-2 cells seeded alone on the COL/FO scaffold, it was observed that the cells continued to proliferate throughout all days of culture time (Fig. 4A). A spheroid structure was first seen in JAR cells on the 7th day of culture, and an increase in spheroids was detected as the culture continued. On the 14th day of culture, it was observed that the spheroids completely covered the surface (Fig. 4B). In the 3D endometrium-like culture system created by seeding RL95-2 and JAR cells onto the COL/FO tissue scaffold, JAR cells formed the first spheroid structures on the 3rd day of culture, and on the 14th day of culture, the spheroids completely covered the surface (Fig. 4C).

**Fig. 5:**
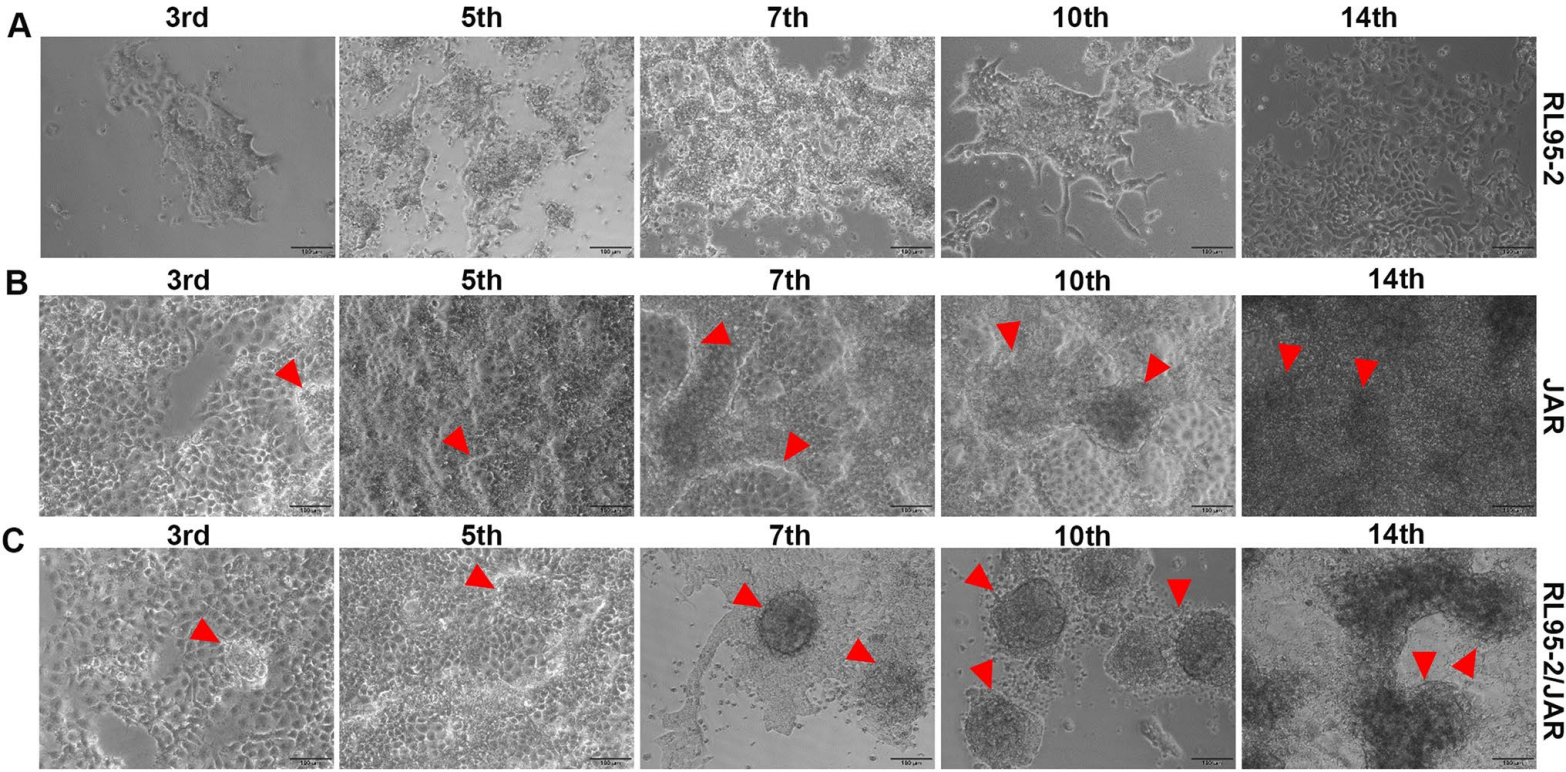
3D endometrium-like culture system created with COL/FI scaffold. **A** Phase-contrast image of RL95-2 cells in COL/FI scaffold from different days of culture. **B** Phase-contrast image of JAR cells in COL/FI scaffold from different days of culture. **C** Phase-contrast image of RL95-2/JAR cells in COL/FI scaffold from different days of culture. The red arrowhead shows spheroids formed by JAR cells. The scale bars:100 μm

In 3D endometrium-like culture systems that was created with COL/FI tissue scaffolding, differences were observed in the cells examined under an inverted microscope on days 3rd, 5th, 7th, 10th, and 14th of cell culture time. Accordingly, in RL95-2 cells seeded alone on the COL/FI scaffold, it was observed that the cells continued to proliferate throughout all days of culture time (Fig. 5A). A spheroid structure was first seen in JAR cells on the 3rd day of culture, and an increase in spheroids was detected as the culture continued. On the 14th day of culture, it was observed that the spheroids completely covered the surface (Fig. 5B). In the 3D endometrium-like culture system that was created by seeding RL95-2 and JAR cells together on the COL/FI tissue scaffold, JAR cells were formed their first spheroid structures on the 3rd day of culture. It was observed that the number of spheroids continued to increase from the 7th day of culture (Fig. 5C).

### Immunocytochemical Findings of a 3D Endometrium-like Culture System Created with BC and COL/FO

#### Compared to the BC Scaffold, the COL/FO Scaffold Better Represents the 3D Endometrium-like Culture System

**Fig. 6:**
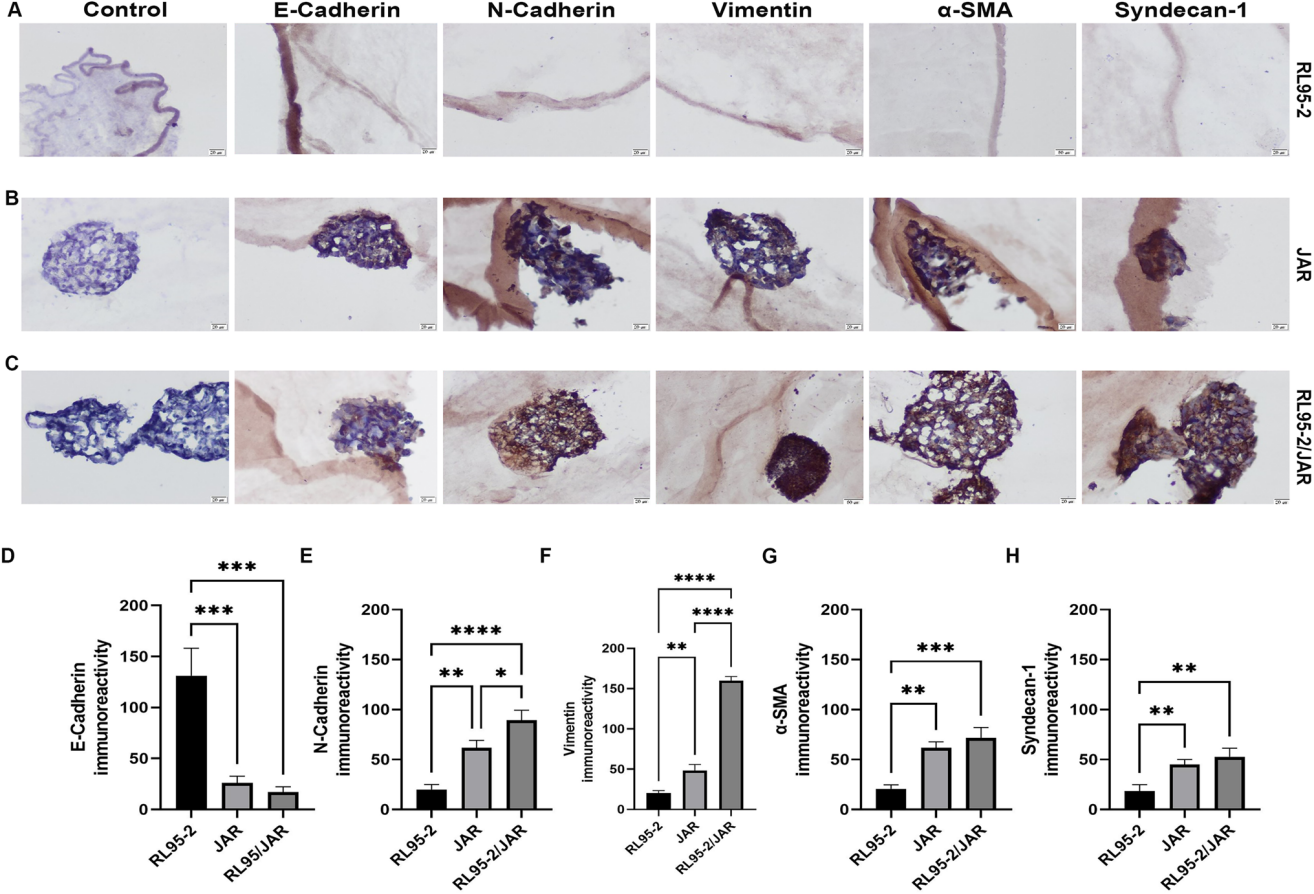
Immunocytochemistry in 3D endometrium-like culture system created with BC tissue scaffold. **A** RL95-2, **B** JAR, **C** RL95-2/JAR cells on control, E-cadherin, N-cadherin, Vimentin, α-SMA, and Syndecan-1 immunocytochemistry staining. The scale bars: 20 μm. Immunoreactivity values of E-cadherin (**D**), N-cadherin (**E**), Vimentin, **F** α-SMA (**G**) , and Syndecan-1 (**H**). Black column represents RL95-2 cells, light grey column represents JAR cells, dark grey column represents co-culture of RL95-2 and JAR cells. Control stains in all groups were negative. Data are presented as mean ± SEM. Statistically significant differences (*p* < 0.05) between all groups were shown using One-Way ANOVA followed by post hoc Tukey multiple comparison test. *P*-values < 0.05 are appended to the graphs. (Represents *p < 0.05, **p < 0.002, ***p < 0.0002, ****p < 0.0001)

**Fig. 7:**
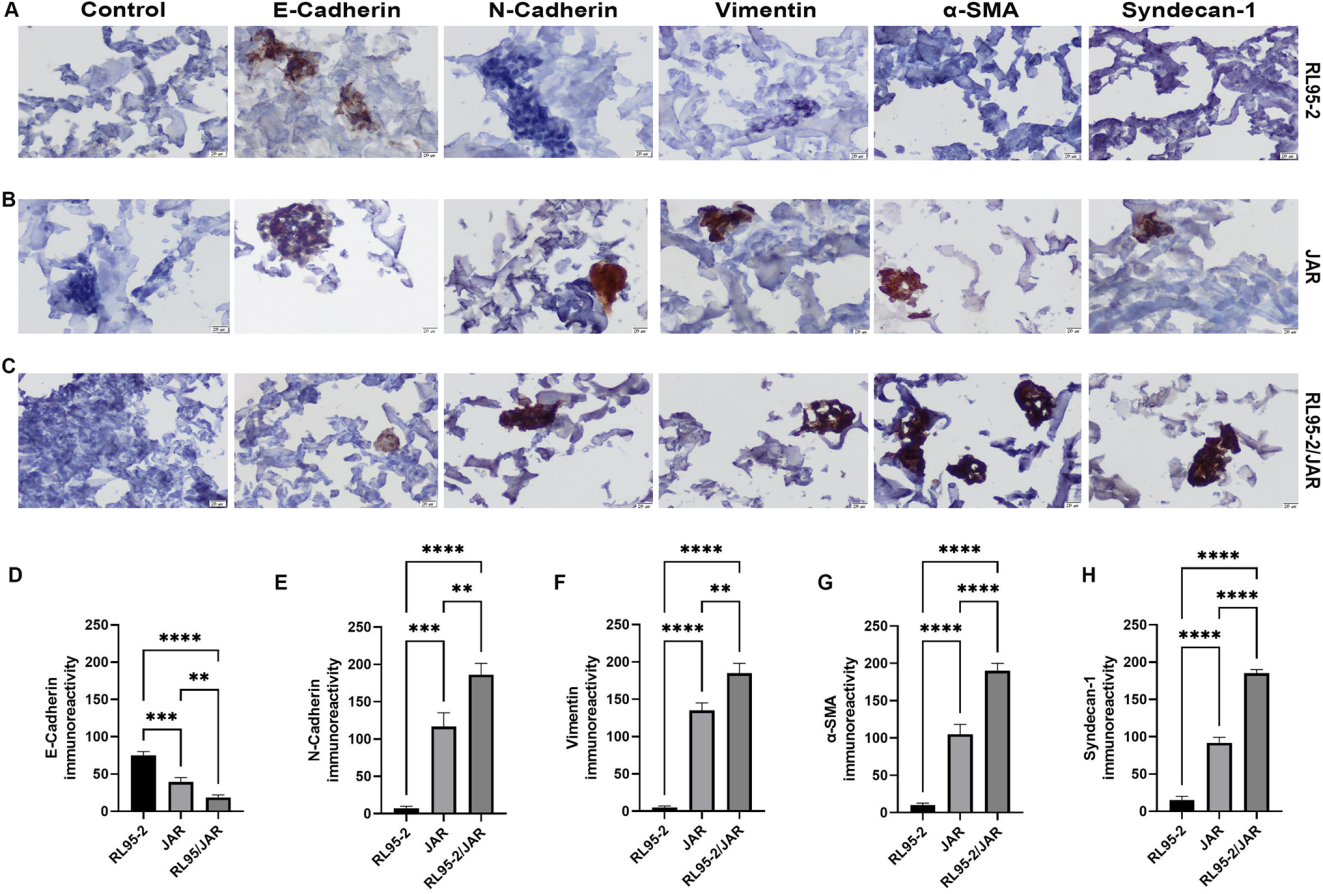
Immunocytochemistry staining in 3D endometrium-like culture system created with COL/FO tissue scaffold. Images represent immunocytochemistry staining of (**A**) RL95-2, **B** JAR, **C** RL95-2/JAR cells with control, E-cadherin, N-cadherin, Vimentin, α-SMA, and Syndecan-1. The scale bars: 20 μm. Immunoreactivity values of E-cadherin (**D**), N-cadherin (**E**), Vimentin, **F** α-SMA (**G**), and Syndecan-1 (**H**). Black column represents RL95-2 cells, light grey column represents JAR cells, dark grey column represents co-culture of RL95-2 and JAR cells. Control stains in all groups were negative. Data are presented as mean ± SEM. Statistically significant differences (*p * < 0.05) between all groups were shown using One-Way ANOVA followed by post hoc Tukey multiple comparison test. *P*-values < 0.05 are appended to the graphs. (Represents *p < 0.05, **p < 0.002, ***p < 0.0002, ****p < 0.0001)

In the 3D endometrium-like culture system, according to the phase-contrast microscope images of the 3rd, 5th, 7th, 10th, and 14th days of cell culture time, the first day of cellular changes in all groups was determined as the 7th day. Accordingly, immunocytochemical analyses of BC and COL/FO tissue scaffolds were compared on day 7th and the differences between the groups were analyzed (Figs. 6 and 7).

Immunocytochemical analyses of RL95-2, JAR and co-cultured RL95-2/JAR cells cultured on BC tissue scaffolds with type-1 epithelial mesenchymal transition markers observed at the invasion step of the implantation process (Fig. 6). E-cadherin immunoreactivity was statistically higher in RL95-2 cells compared to JAR and RL95-2/JAR cells (*p* < 0.002). However, there was no statistically significant difference between JAR and RL95-2/JAR cells (*p* > 0.05) (Fig. 6D). N-cadherin immunoreactivity was statistically higher in JAR and RL95-2/JAR cells compared to RL95-2 cells (*p* < 0.002 and *p* < 0.0001, respectively). Similarly, N-cadherin immunoreactivity was statistically higher in RL95-2/JAR cells compared to JAR cells (*p* < 0.05) (Fig. 6E). Vimentin immunoreactivity was statistically higher in JAR and RL95-2/JAR cells compared to RL95-2 cells (*p* < 0.002 and *p* < 0.0001, respectively). Furthermore, Vimentin immunoreactivity was statistically higher in RL95-2/JAR cells compared to JAR cells (*p* < 0.0001) (Fig. 6F). α-SMA immunoreactivity was statistically higher in JAR and RL95-2/JAR cells compared to RL95-2 cells (*p* < 0.002 and *p* < 0.0002, respectively). However, there was no statistically significant difference between JAR and RL95-2/JAR cells (*p* > 0.05) (Fig. 6G). Syndecan-1 immunoreactivity was statistically higher in JAR and RL95-2/JAR cells compared to RL95-2 cells (*p* < 0.002). However, there was no statistically significant difference between JAR and RL95-2/JAR cells (*p* > 0.05) (Fig. 6H).

Immunocytochemical analyses of RL95-2, JAR and co-cultured RL95-2/JAR cells cultured on COL/FO tissue scaffolds with type-1 epithelial mesenchymal transition markers observed at the invasion step of the implantation process (Fig. 7). E-cadherin immunoreactivity was statistically higher in RL95-2 cells compared to JAR and RL95-2/JAR cells (*p* < 0.002 and *p* < 0.001, respectively). Furthermore, E-cadherin immunoreactivity was also statistically higher in JAR cells compared to RL95-2/JAR cells (*p* < 0.002) (Fig. 7D). N-cadherin immunoreactivity was statistically higher in JAR and RL95-2/JAR cells compared to RL95-2 cells (*p* < 0.002 and *p* < 0.0001, respectively). Similarly, N-cadherin immunoreactivity was statistically higher in RL95-2/JAR cells compared to JAR cells (*p* < 0.002) (Fig. 7E). Vimentin immunoreactivity was statistically higher in JAR and RL95-2/JAR cells compared to RL95-2 cells (*p* < 0.0001). Furthermore, Vimentin immunoreactivity was statistically higher in RL95-2/JAR cells compared to JAR cells (*p* < 0.002) (Fig. 7F). α-SMA immunoreactivity was statistically higher in JAR and RL95-2/JAR cells compared to RL95-2 cells (*p* < 0.0001). Similarly, α-SMA immunoreactivity was statistically higher in RL95-2/JAR cells compared to JAR cells (*p* < 0.0001) (Fig. 7G). Syndecan-1 immunoreactivity was statistically higher in JAR and RL95-2/JAR cells compared to RL95-2 cells (*p* < 0.0001). Furthermore, Syndecan-1 immunoreactivity was statistically higher in RL95-2/JAR cells compared to JAR cells (*p* < 0.0001) (Fig. 7H).

### Double Immunofluorescence Findings of a 3D Endometrium-like Culture System Created with COL/FI

#### COL/FI Scaffold Perfectly Supports the Topographic Features of the Cells in the 3D Endometrium-like Culture System

In the 3D endometrium-like culture system, according to the phase-contrast microscope images of the 3rd, 5th, 7th, 10th, and 14th days of cell culture time, the first day of cellular changes in all groups was determined as the 7th day. Accordingly, immunofluorescence analyses of COL/FI tissue scaffolds were compared on day 7th and the differences between the groups were analyzed.

**Fig. 8:**
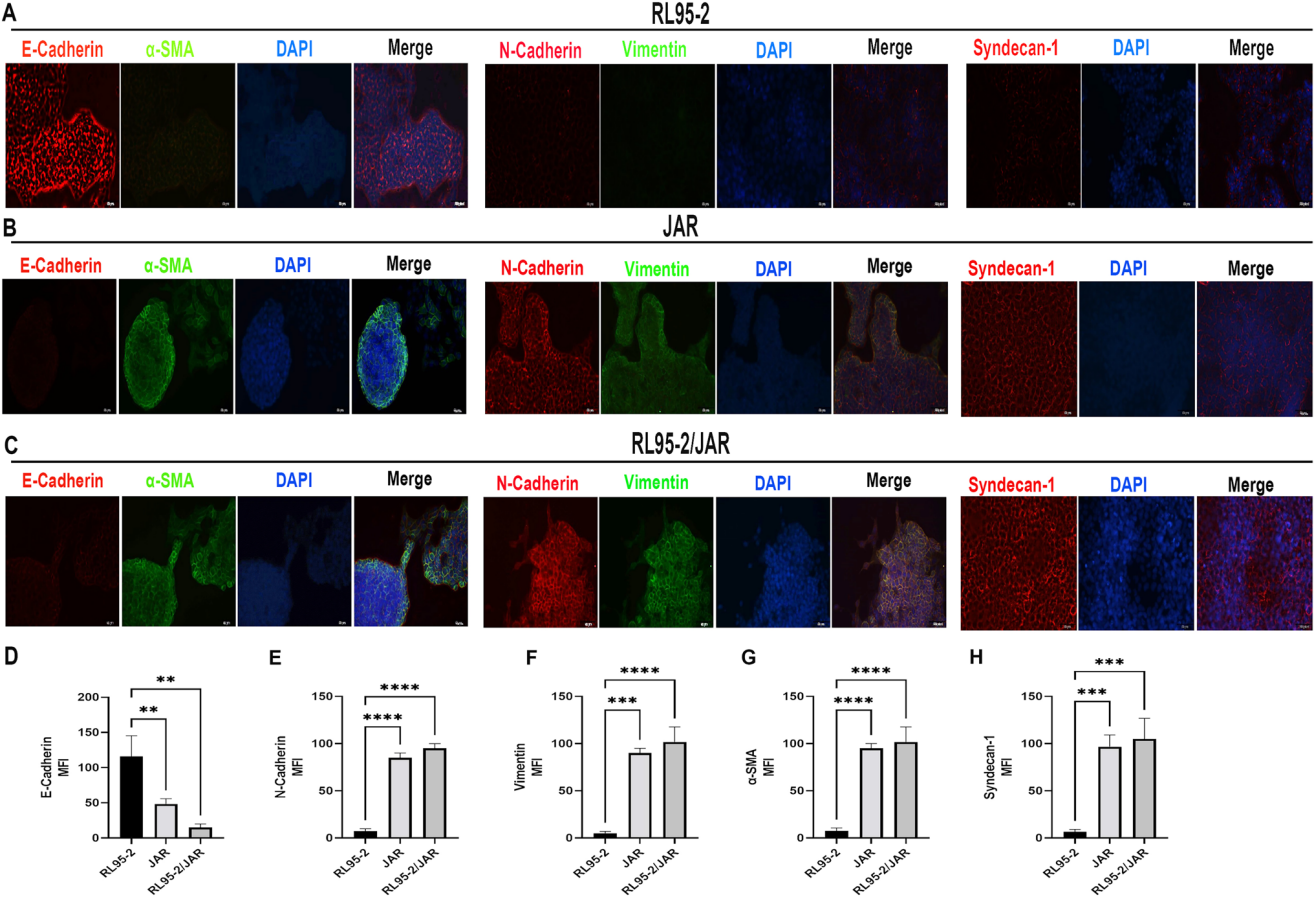
Immunofluorescence staining in 3D endometrium-like culture system created with COL/FI tissue scaffold. staining. Images represent immunofluorescence staining of (**A**) RL95-2, **B** JAR, **C** RL95-2/JAR cells with E-cadherin, N-cadherin, and Syndecan-1 (Rodamine-red), Vimentin and α-SMA (FITC-green) and counterstaining of nuclei with DAPI dye (blue). The scale bars: 200 μm. Mean fluorescence intensity (MFI) values of E-cadherin (**D**), N-cadherin (**E**), Vimentin, **F** α-SMA (**G**), and Syndecan-1 (**H**). Black column represents RL95-2 cells, light grey column represents JAR cells, dark grey column represents co-culture of RL95-2 and JAR cells. Data are presented as mean ± SEM. Statistically significant differences (*p* < 0.05) between all groups were shown using One-Way ANOVA followed by post hoc Tukey multiple comparison test. *P*-values < 0.05 are appended to the graphs. (Represents **p < 0.002, ***p < 0.0002, ****p < 0.0001)

Immunofluorescence analyzes of RL95-2, JAR, and co-cultured RL95-2/JAR cells cultured on COL/FI scaffolds with type-1 epithelial-mesenchymal transition markers observed during the invasion phase of the implantation process (Fig. 8). Mean fluorescence intensity (MFI) of E-cadherin was statistically higher in RL95-2 cells than in JAR and RL95-2/JAR cells (*p* < 0.002). However, this immunofleurosence staining was no statistically significant difference between JAR and RL95-2/JAR cells (*p* > 0.05) (Fig. 8D) Double immunofluorescence findings N-cadherin MFI was statistically higher in JAR and RL95-2/JAR cells compared to RL95-2 cells (*p* < 0.0001). However, there was no statistically significant difference between JAR and RL95-2/JAR cells (*p* > 0.05) (Fig. 8E). Vimentin MFI was statistically higher in JAR and RL95-2/JAR cells than in RL95-2 cells (*p* < 0.0002 and *p* < 0.0001, respectively). However, there was no statistically significant difference between JAR and RL95-2/JAR cells (*p* > 0.05) (Fig. 8F). α-SMA MFI was statistically higher in JAR and RL95-2/JAR cells compared to RL95-2 cells (*p* < 0.0001). However, there was no statistically significant difference between JAR and RL95-2/JAR cells (*p* > 0.05) (Fig. 8G). Syndecan-1 MFI was statistically higher in JAR and RL95-2/JAR cells than in RL95-2 cells (*p* < 0.0002). However, there was no statistically significant difference between JAR and RL95-2/JAR cells (*p* > 0.05) (Fig. 8H).

## Discussion

Embryo implantation, the first step in establishing a healthy pregnancy, involves the interaction of an invasive embryo and a receptive endometrium. Defects in the invasion steps of trophoblast cells during embryo implantation have been shown to contribute to pregnancy complications such as implantation failure and spontaneous abortion [10]. Considering the difficulties in obtaining human primary cells, lack of sufficient human samples and culture problems, immortalized cell lines are preferred in in vitro studies [11].

In the 3D culture model created with collagen tissue scaffold, single cultivation of RL95-2 cells and co-culture model created with human endometrial stromal cells, differences were found in the invasion of JAR cells. Accordingly, in the co-culture model of RL95-2 cells with endometrial stromal cells, necrotic structures were observed in epithelial cells after invasion of JAR spheroids. However, in the model in which JAR spheroids were co-cultured only with RL95-2 cells, invasion of JAR spheroids was found to be more successful [12].

Epithelial mesenchymal transition (EMT) occurs in physiological events such as embryo implantation and pathological events such as cancer, where cells undergo structural and biochemical changes [13]. Elucidation of the mechanism of type 1 epithelial mesenchymal transition in implantation, embryogenesis and organ development may contribute to the understanding of normal and abnormal pregnancy cases. Cell surface proteins such as E-cadherin, N-cadherin, Syndecan-1 and cytoplasmic proteins such as Vimentin and α-SMA are involved in both embryo implantation and cancer [2].

Since many pregnancy complications or spontaneous abortion are associated with implantation, 3D functional endometrium-like culture systems to be established to elucidate the mechanism of implantation are very important [14].However, the development of scaffolds that best mimic the endometrium, allow cellular invasion, are biodegradable and biocompatible still needs to be extensively investigated [15].

In our study, BC, COL/FO and COL/FI were chosen because they are easy to produce, have low costs, do not show toxic effects on cells, can be analyzed, and support the proliferation and differentiation of cells. A three-dimensional endometrium-like culture system was established by adding RL95-2 cells, an endometrial epithelial cell line, and JAR cells, a trophoblast-like choriocarcinoma cell line with the ability to form spheroids, to these three different tissue scaffolds. Quantitative analyses of E-cadherin, N-cadherin, Vimentin, α-SMA and Syndecan-1, which are involved in the type 1 epithelial mesenchymal transition mechanism seen in the invasion step of the implantation process, were performed in the established endometrium-like culture systems.

BC is a high-purity biocompatible natural polymer with a fine fiber network containing type 1 collagen [15, 16]. Compared to 2D culture results, BC caused a delay in the formation of JAR spheroids, suggesting that perhaps it is not sufficient support material for JAR spheroids. However, it was observed that spheroid formation was accelerated in the 3D endometrium-like culture system formed by co-culturing RL95-2 and JAR cells on BC tissue scaffold. This result indicated that paracrine interactions between RL95-2 and JAR cells were healthy under the influence of 3D culture induced by BC.However, considering that there was a difference only in N-cadherin and Vimentin immunoreactivity in RL95-2/JAR cells compared to JAR cells and no difference in other mesenchymal cell markers, it was concluded that BC partially mimics the 3D endometrium-like culture system.

COL/FO and COL/FI scaffolds are biomaterials with randomly arranged collagen fibers similar to in vivo, which support the proliferation and invasion of cells and enable the differentiation of cells in the targeted functional tissue. In the 3D endometrium-like culture system created by co-culturing RL95-2 and JAR cells on COL/FO tissue scaffold, it was shown that RL95-2 cells accelerated the spheroid formation in JAR cells. This result indicated that the paracrine interactions between RL95-2 and JAR cells under 3D culture with COL/FO were healthy.In the COL/FO-induced 3D culture model, low immunoreactivity of E-cadherin, a marker of epithelial cells, and high immunoreactivity of N-cadherin, Vimentin, α-SMA and Syndecan-1, markers of mesenchymal cells, in JAR and RL95-2/JAR cells indicated that COL/FO supported invasion of cells. However, the statistically higher immunoreactivity of all mesenchymal cell markers in RL95-2/JAR cells compared to JAR cells indicated that COL/FO supported cell invasion better than BC scaffold and mimicked 3D endometrium-like culture system better.

When compared with 2D culture results, it suggested that COL/FI caused acceleration in the formation of JAR spheroids and thus supported the formation of JAR spheroids. In the 3D endometrium-like culture system formed by co-culturing RL95-2 and JAR cells on COL/FI tissue scaffold, the first spheroid formation was observed on day 3 and the number of spheroids continued to increase from day 7. This result showed that both COL/FI scaffold accelerated the spheroid formation in JAR cells and supported the topographic features of the cells. The spheroid diameter of JAR cells in the COL/FI scaffold is measurable and numerous. Therefore, it was suggested that COL/FI scaffold is a biomaterial capable of exhibiting the topographical features of cells.

In the COL/FI-induced 3D culture model, low immunoreactivity of E-cadherin, a marker of epithelial cells, and high immunoreactivity of N-cadherin, Vimentin, α-SMA and Syndecan-1, markers of mesenchymal cells, in JAR and RL95-2/JAR cells indicated that COL/FI supported invasion of cells. However, all mesenchymal markers were similar between RL95-2/JAR and JAR cells, indicating that the COL/FI scaffold could not mimic the 3D endometrium-like culture system as well as either COL/FO or BC. However, given that COL/FI scaffold supports the topography of the cells very well, unlike COL/FO and BC, we think that COL/FI scaffolds should be used to support the topography of the cells and COL/FO scaffolds should be used in combination to support invasion for future 3D endometrium-like culture systems.

## Conclusion

This study investigated in detail the cell behaviour in 3D endometrium-like culture systems using BC, COL/FO and COL/FI scaffolds with endometrial epithelial cell line RL95-2 and spheroid-forming trophoblast-like choriocarcinoma cell line JAR cells. BC tissue scaffold promoted the proliferation of RL95-2 cells, whereas it delayed the formation of JAR spheroids. COL/FO scaffold promoted the proliferation of RL95-2 cells and accelerated the formation of JAR spheroids. COL/FI scaffold is important in terms of supporting the topography of the cells and may offer a potential solution when combined with COL/FO. Immunocytochemical and immunofluorescence analyses showed that scaffolds modulate the invasion process by affecting the expression of EMT proteins in cells. These findings suggest that different scaffolds may produce different effects in endometrium-like culture systems and that combinations of these materials may yield more effective results in future studies. This research provides a basic guideline for understanding and modifying cell behaviour in 3D culture systems.

**Fig. 9:**
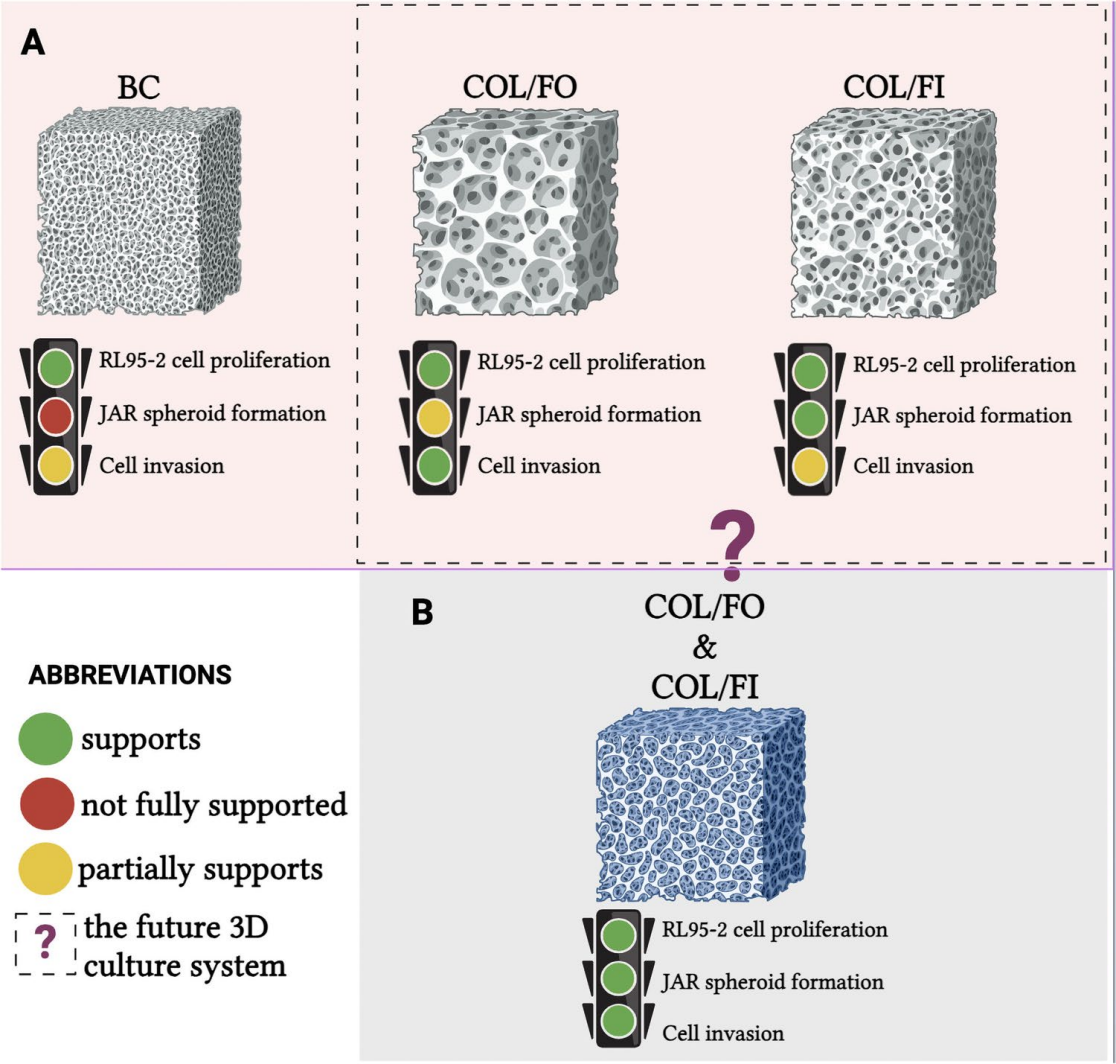
Graphical summary of our work. The figure was created with Biorender software

This study has not only gone beyond a scientific understanding with the findings obtained in 3D endometrium-like culture systems created using three different tissue scaffolds, but also shed new light on future biomedical research. This study has taken a critical step forward in the study and understanding of cell behavior, particularly elucidating the complexity of the endometrium and the mechanisms of invasion in detail. Furthermore, understanding the effects of different scaffolds on cell function has paved the way for the development of new strategies in regenerative medicine and cellular therapy. These findings may contribute to the development of more effective and customized therapeutic approaches by providing advanced guidance for bioengineering and medical researchers, especially in the development of 3D culture systems and the selection of biomaterials. In conclusion, this research is an important contribution to the scientific community with its far-reaching potential to understand cell-matrix interactions, to create new therapeutic strategies and to take important steps in regenerative medicine. Figure 9 summarises the result of our study.

## Data Availability

Data supporting the findings of this study are available from the corresponding author upon reasonable request.

## Code Availability

Not applicable, as no software or custom code was developed for this study.
